# Supplementary material for: A 33-mRNA Classifier Is Able to Produce Inflammopathic, Adaptive, and Coagulopathic Endotypes with Prognostic Significance: The Outcomes of Metabolic Resuscitation Using Ascorbic Acid, Thiamine, and Glucocorticoids in the Early Treatment of Sepsis (ORANGES) Trial
Source: J Pers Med. 2020 Dec 23;11(1):9. doi: 10.3390/jpm11010009 (PMC7822486; doi:10.3390/jpm11010009)
Supplement: Supplementary file 1 [file jpm-11-00009-s001.pdf]

Supplemental Fig. 1)

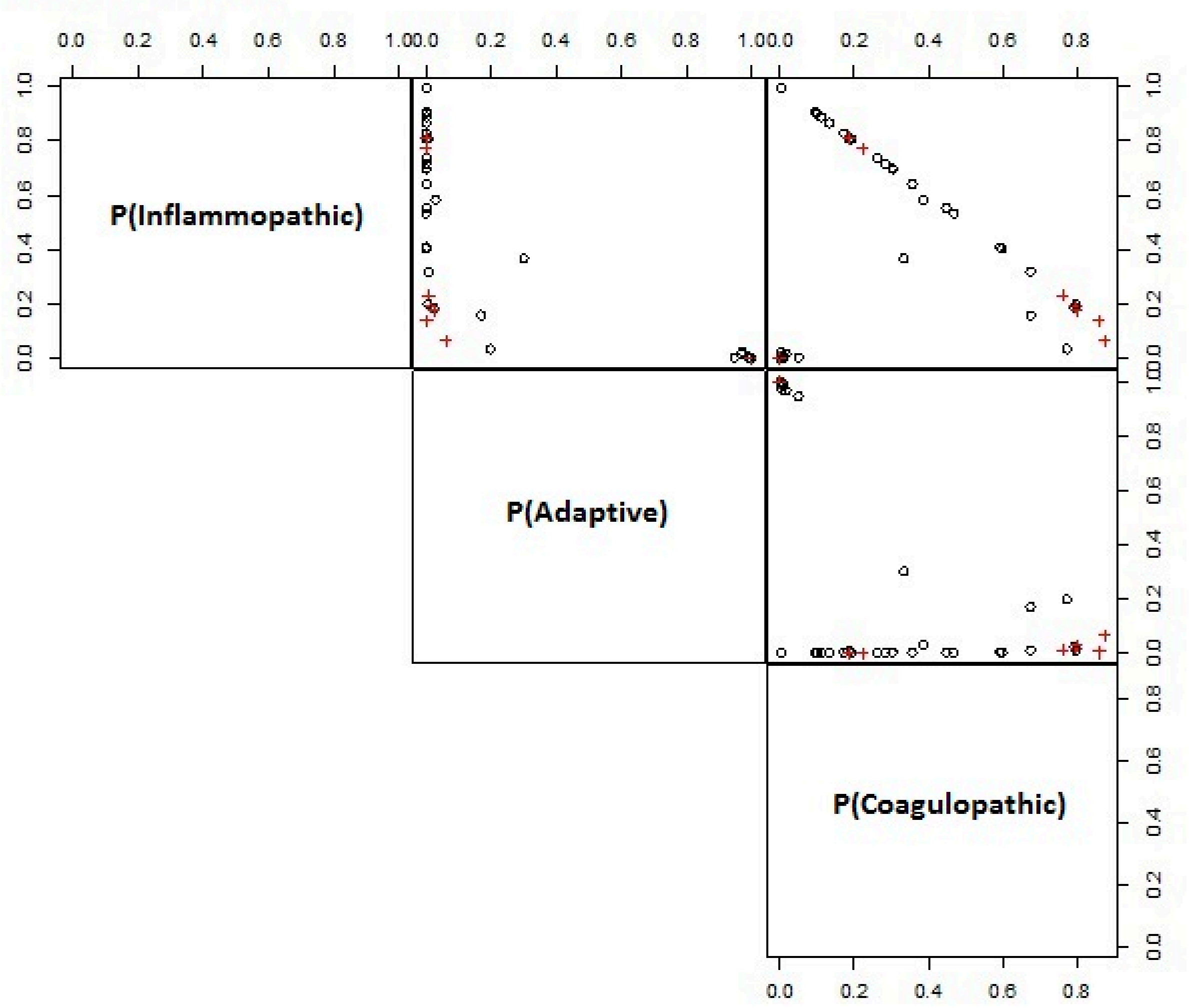

Supplemental Figure 1. Probabilities of endotype assignment as produced by the multivariable classifier. Note that for each sample, the sum of p(Inflammopathic) + p(Adaptive) + p(Coagulopathic) = 1. Few subjects have a 'mid-range' Adaptive score. As shown in the upper-right box, there is a wide spectrum of Inflammopathic-Coagulopathic probabilities, indicating some difficulty in distinguishing between these two with the current classifier. Endotype was assigned to highest probability class for each sample. Red plus signs represent patients who died.

Supplemental Fig. 2)

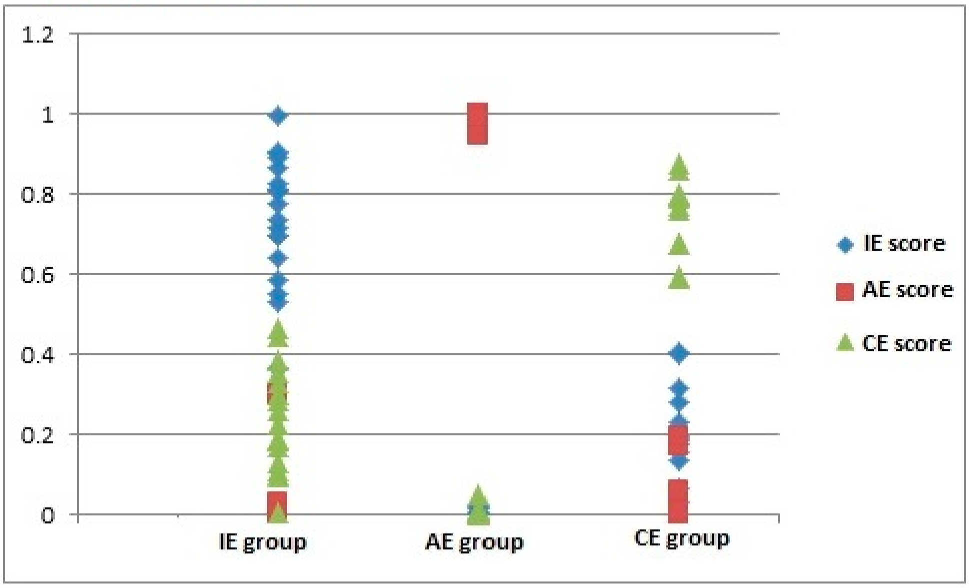

Supplemental Figure 2) Probabilities of endotype assignment as produced by the multivariable classifier grouped according to final assignment. For each sample, the three probabilities are depicted: pIE (blue), pAE (red), and pCE (green). Note that the sum of pIE + pAE + pCE = 1. Although separation is least between the IE and CE groups, all pIE exceed all pCE in samples assigned to the IE group, and vice versa, consistent with the validity of endotype assignment.
